# Intake of 100% Fruit Juice Is Associated with Improved Diet Quality of Adults: NHANES 2013–2016 Analysis

**DOI:** 10.3390/nu11102513

**Published:** 2019-10-18

**Authors:** Sanjiv Agarwal, Victor L. Fulgoni III, Diane Welland

**Affiliations:** 1 NutriScience LLC, East Norriton, PA 19403, USA; 2 Nutrition Impact LLC, Battle Creek, MI 49014, USA; vic3rd@aol.com; 3 Juice Product Association, Washington, DC 20045, USA; DWelland@kellencompany.com

**Keywords:** nutrients, healthy eating index, health risk factors, dietary modeling

## Abstract

Fruit intake is generally associated with better diet quality and overall health. This report examined the effect of 100% fruit juice (considered a part of total fruit servings) and its replacement with whole fruits equivalents on nutrient intake and diet quality. National Health and Nutrition Examination Survey 2013–2016 data (24-h dietary recall) from adults 19+ years (*n* = 10,112) were used to assess the diet quality and nutrient intakes and to isocalorically replace with 100% fruit juice intakes whole fruit equivalents in a modeling analysis. About 15.6% adults were 100% fruit juice consumers. Consumers had higher diet quality (10% higher Healthy Eating Index, HEI 2015 score), and higher intakes of energy, calcium, magnesium, potassium, vitamin C and vitamin D than non-consumers. Consumption of 100% fruit juice was also associated with lower risk of being overweight/obese (−22%) and having metabolic syndrome (−27%). Replacing 100% fruit juice with whole fruits equivalents did not affect nutrient intake except for a modest increase (+6.4%) in dietary fiber. Results show that 100% fruit juice intake was associated with better diet quality and higher nutrient intake. Replacement of 100% fruit juice intake with whole fruits equivalents had no significant effect on nutrients except for dietary fiber.

## 1. Introduction

Increased fruit and vegetable consumption are associated with a reduced risk of CVD, diabetes and stroke, and their low intake is linked with poor health and increased risk of chronic diseases [1,2,3,4,5]. Fruits and vegetables are important sources of a number of key nutrients, including K, Mg, dietary fiber, folate, and vitamins A and C, and an array of bioactive substances [1,6,7,8]. Due to their nutritional value, they are consistently recommended by public health authorities globally and increasing their consumption is an important public health goal [9]. Dietary Guidelines for Americans 2015–2020 (DGA) recommends consumption of fruits and vegetables as part of healthy eating pattern [1]. Two or more servings of fruits and three or more servings of vegetables per day are recommended by most nutritional guidelines [2]. Increasing the contribution of fruits to the diets of adults and children is also one of the key objectives of Healthy People 2020 [10]. Despite these recommendations, there is a huge gap between recommendations and consumption. In 2015, only 12.2% U.S. adults (9.2% male and 15.1% females) met fruit intake recommendations and 9.3% adults (7.6% male and 10.9% females) met vegetable intake recommendations [11]. 

ChooseMyPlate recommends that half of the food on a meal plate should be fruit and vegetables and adults should consume 1.5 to 2 cups equivalent fruit per day depending on age, gender, and physical activity [6]. The fruit requirement can be met by consuming fresh, frozen, or dried whole fruit, or 100% fruit juice. DGA and MyPlate indicated that one cup of 100% fruit juice can be considered as one cup serving from the Fruit Group and 100% fruit juice in moderation can be part of healthy eating patterns [1,6]. However, there is an ongoing scientific debate on the recommendations for 100% fruit juice intake, especially for children. Concerns have been raised that naturally occurring sugars in 100% fruit juices may cause weight gain similar to those of sugar-sweetened beverages, again especially in children [12,13,14], however, several studies concluded that 100% fruit juice was not associated with meaningful weight gain [14,15,16]. Several randomized controlled trials have also suggested a positive or null effect of 100% fruit juice on cardiometabolic risk factors and glucose control [17,18]. A few previous cross-sectional studies also reported that children and adults who consumed 100% fruit juice had better diet quality and nutrient intakes than non-consumers [19,20,21,22,23,24,25]. The main purpose of this study was to provide an updated evaluation of the association of 100% fruit juice consumption by consumption level and the effect of replacing 100% fruit juice with whole fruit equivalents on nutrient intake and diet quality using the most recent National Health and Nutrition Examination Survey (NHANES) 2013–2016 database. Secondary aim of this study was to evaluate the association of 100% fruit juice consumption with physiological markers of risk. 

## 2. Methods

### 2.1. Data Collection

The NHANES is a cross-sectional survey of nationally-representative non-institutionalized civilian population conducted by the National Center for Health Statistics (NCHS) of the Centers for Disease Control and Prevention (CDC) on a continual basis to examine nutrition, diet and health relationship. The data are collected using a complex stratified multistage cluster sampling probability design via an in-home interview for demographic and basic health information, and a comprehensive diet and health examination in a mobile examination center. A detailed description of the subject recruitment, survey design, and data collection procedures are available online [26] and all data obtained from this study are publicly available at: http://www.cdc.gov/nchs/nhanes/. NHANES protocol was approved by the NCHS Ethics Review Board and all participants or proxies provided a signed written informed consent. This study was a secondary data analysis which lacked personal identifiers, therefore, did not require Institutional Review Board review.

### 2.2. Study Population

Data from adults age 19+ years participating in NHANES 2013–2014, and 2015–2016 (*n* = 11,776) were used; however, those with unreliable data (*n* = 1461), primarily incomplete recalls, determined by the United States Department of Agriculture (USDA) and pregnant or lactating females (*n* = 203) were excluded, and the final sample size was 10,112 adults. 

### 2.3. Estimates of Dietary Intake 

Dietary intake data were obtained from in-person 24-h dietary recall interviews that were administered using an automated, multiple-pass (AMPM) method [27]. While two dietary recalls were collected the first day dietary recall was collected with methods that have been validated and as such only this dietary recall was used in all analyses. 100% fruit juice intakes were assessed from 30 available USDA food codes beginning with 612 and 614 (Table 1). Fruit juices reconstituted from concentrate with water were also considered as100% fruit juice. Juice cocktails, juice punches, juice drinks, or juice beverages and fruit juices with any added sugars were not considered as 100% fruit juice in this study. Fruit juice consumers were defined as those consuming any amount of 100% fruit juice during the first 24-h recall. Participants were dichotomized into consumers and non-consumers of 100% fruit juice; and consumers were further classified into 4 groups based on 100% fruit juice consumption levels:(>0–4 oz, >4–8 oz, >8–12 oz and >12 oz. Energy and nutrient intake were determined by using the USDA Nutrient Database for Standard Reference Releases in conjunction with the respective Food and Nutrient Database for Dietary Studies for each NHANES cycle [28,29]. 

### 2.4. Estimates of Diet Quality 

Diet quality scores were determined using the USDA Healthy Eating Index-2015 (HEI-2015) [30]. The HEI-2015 contains 13 subcomponents, each reflecting the DGA’s recommendations. Dietary intake was expressed per 1000 kilocalories for all components except for fatty acid ratios (expressed as ratio of unsaturated to saturated fatty acids), saturated fat (expressed as % energy) and added sugars (expressed as % energy). Total vegetables; greens and beans; total fruit, whole fruit; total protein; and seafoods and plant proteins were scored proportionally from 0 to 5 points and all other components (i.e., whole grains; dairy; fatty acids; sodium; refined grains; saturated fat; and added sugars) were scored proportionally from 0 to 10 points. Four components, sodium, refined grains, saturated fat, and added sugars are reverse scored, so that lower intake leads to a higher score, and thus a greater contribution to overall diet quality. The maximum possible score was 100 [30].

### 2.5. Estimation of Physiological Markers of Risk 

Body weight, body mass index (BMI), waist circumference, blood pressure, total cholesterol, LDL-cholesterol (fasting), HDL-cholesterol, triglycerides (fasting), plasma glucose (fasting), glycohemoglobin, and insulin (fasting) were measured using NHANES standard protocols [26]. Homeostasis model assessment: insulin resistance (HOMA-IR) was calculated as: insulin (mU/L) × plasma glucose (mmol/L)/22.5 [31]. The following criteria were used to define risk factors: elevated waist circumference: waist circumference > 102 cm for males, >88 cm for females; elevated blood pressure: systolic BP ≥ 130 mmHg or diastolic BP ≥ 80 mmHg or taking hypertension medication; reduced HDL-cholesterol: HDL-cholesterol < 40 mg/dL for males, <50 mg/dL for females or taking antihyperlipidemic medication; elevated triglycerides: triglycerides ≥ 150 mg/dL or taking antihyperlipidemic medication; elevated plasma glucose: plasma glucose > 100 mg/dL or taking antidiabetic medication; metabolic syndrome: positive diagnosis for 3 or more of the risk factors described above; overweight or obese: BMI ≥ 25 kg/m^2^; elevated LDL-cholesterol: LDL ≥ 100 mg/dL or taking antihyperlipidemic medication [32,33]. 

### 2.6. Dietary Modeling 

Intake 100% fruit juice in consumers was isocalorically replaced by whole fruit equivalents (food codes beginning with 611, 631, 632 and 633; Table 1) in the juice modeling analysis. Usual intakes (UI) of nutrients was estimated using the National Cancer Institute (NCI) Method V. 2.1 [34]; the percentage of the population below the Estimated Average Requirement (EAR) or above Adequate Intake (AI) were estimated with two days of intake data in 100% fruit juice consumers before and after replacement. 

### 2.7. Statistics 

All analyses were performed using SAS 9.4 (SAS Institute, Cary, NC, USA) software. The data were adjusted for the complex sampling design of NHANES, using appropriate survey weights, strata, and primary sampling units. Day1 dietary/examination weights were used in all analysis except where the outcome was a fasting laboratory variable in which case fasting subsample weights were used. 

Mean descriptive data were determined for consumers and non-consumers of 100% fruit juice; differences in groups were determined via *t*-tests. Least square means (LSM) and standard errors (SE) were generated *via* regression analyses for energy and nutrient intakes; diet quality; and physiological risk markers in non-consumers and 100% fruit juice consumers (including consumers by consumption level). Analyses were adjusted for age, gender, ethnicity, physical activity level, poverty income ratio level, current smoking status, alcohol and energy intake (except for energy and diet quality) for energy, nutrients and diet quality. BMI was also added to covariate list for all physiological and risk variables except for body weight, BMI, waist circumference, overweight or obese status, elevated waist circumference status and metabolic syndrome. The p-values for trend across fruit juice consumption level in the LSM and odds ratios (OR) analyses were based on models with 100% fruit juice (oz) as a continuous variable. Significant differences before and after isocaloric replacement of 100% fruit juice intakes by whole fruit equivalents in modeling analysis were accessed by a Z-statistic being compared to a normal distribution table.

## 3. Results

### 3.1. Demographics 

Approximately 15.6% of adults consumed 100% fruit juice and about 1.2%, 4.6%, 4.2%, and 5.7% of adults consumed > 0 to 4 oz/day, >4 to 8 oz/day, >8 to 12 oz/day, and >12 oz/day, respectively. Adult consumers of 100% fruit juice were older, and had lower BMI compared to non-consumers (*P* < 0.05). A significantly higher proportion of 100% fruit juice consumers were male, Hispanic, non-Hispanic blacks, and lower proportion were non-Hispanic white, of other ethnicity, smokers, obese compared to non-consumers (*P* < 0.05). All other demographic characteristics evaluated were similar among consumers and non-consumers of 100% fruit juice (Table 2).

### 3.2. 100% Fruit Juice Intake 

Per capita mean usual intake (a measure of long-term intake) of 100% fruit juice was 0.26 ± 0.01 cups eq/day with a 95th percentile of intake of 1.11 cups eq/day. 100% fruit juice provided on average of 153 ± 4 kcal/day or 7% energy, 138 ± 6 mg/day or 14% calcium, 30.3 ± 0.7 mg/day or 10% magnesium, 480 ± 11 mg/day or 16% potassium, 94.4 ± 1.9 mg/day or 61% vitamin C, 0.83 ± 0.02 g/day or 5% dietary fiber and 29.1 ± 0.7 g/day or 23% total sugars (by definition 100% fruit juice provides zero added sugars) to the consumers on the day of recall.

### 3.3. Effect of Intake of 100% Fruit Juice on Energy and Nutrients Intake 

There were significant differences in energy and nutrient intakes between the 100% fruit juice consumers and the non-consumers (Table 3). Consumers had a significantly higher intake of energy (+8.3%) and energy adjusted carbohydrates (+8.6%), total sugar (+18.1%), calcium (+8.0%), magnesium (+3.3%), potassium (+13.2%), thiamin (+5.1%), folate (+10.1%), vitamins B6 (+6.6%), vitamin C (+143%), Vitamin D (+17.8%) and beta-cryptoxanthin (+70.7%), and lower intakes for added sugars (−14.5%), total fat (−8.9%), protein (−3.9%) and sodium (−4.4%) compared to non-consumers. The intakes of energy, carbohydrates, total sugars, calcium, magnesium, potassium, folate, vitamin B6, vitamin C, vitamin D and beta-cryptoxanthin also increased while the intakes of added sugars, total fat, protein, sodium, riboflavin, niacin, decreased with increasing 100% fruit juice consumption level (Table 3).

### 3.4. Effect of Intake of 100% Fruit Juice on Diet Quality 

Adult consumers of 100% fruit juice as compared to non-consumers had a 5.0 point or 10% higher (*P* < 0.0001) HEI-2015 (a measure of diet quality) total score and there was a significant group trend (*P* < 0.0001) for increasing HEI-2015 total score with increasing consumption level (Table 4). The HEI 2015 total score of adult consumers were also significantly higher compared to non-consumers when the data was analyzed separately for males and females and for age groups 19–30, 19–50, 31–50, 51–70, 51–99 and 71–99 years (data not presented). The HEI 2015 subcomponent scores for total fruit, whole fruit, whole grain, sodium, saturated fat and added sugar were also significantly higher (*P* < 0.05 for whole grain and *P* < 0.01 for other variables) for consumers compared to non-consumers with a significant group trend (*P* < 0.01) for increasing HEI-2015 subcomponent scores (total fruit, whole fruit, sodium, saturated fat, and added sugar) with increasing 100% fruit juice consumption level (Table 4). 

### 3.5. Effect of Intake of 100% Fruit Juice on Physiological Markers 

100% fruit juice adult consumers as compared to non-consumers had a significantly lower BMI (28.3 ± 0.3 vs. 29.5 ± 0.2 kg/m^2^, *P* = 0.0009), body weight (80.4 ± 1.0 vs. 84.0 ± 0.5 kg, *P* = 0.0019), waist circumference (97.9 ± 0.8 vs. 101 ± 0.3 cm, *P* = 0.0025), plasma glucose 106 ± 1 vs. 109 ± 1 mg/dL, *P* = 0.0491), and glycohemoglobin (5.59 ± 0.03% vs. 5.68 ± 0.01%, *P* = 0.0035). Adult consumers of 100% fruit juice also had a significantly lower risk for being overweight or obese (OR = 0.78; 95% CI = 0.65, 0.95; *P* = 0.0147), having an elevated waist circumference (OR = 0.69; 95% CI = 0.56, 0.85; *P* = 0.0012) and metabolic syndrome (OR = 0.73; 95% CI = 0.58, 0.93; *P* = 0.0115) as compared to non-consumers.

### 3.6. Effect of Isocaloric Replacement of 100% Fruit Juice with Whole Fruit Equivalents

When 100% fruit juice was isocalorically replaced by whole fruit equivalents, there was a significant increase (+6.4%, *P* = 0.0008) in usual intake of fiber (Table 5) for consumers. There was also a significant increase (*P* = 0.0102) % of population with intakes above AI for dietary fiber with replacement. However, the replacement did not significantly affect (*P* > 0.05) usual intake or inadequacy (% population below EAR) or % population above AI for any other nutrients (Table 5). 

## 4. Discussion

In the present analysis of NHANES 2013–2016 using the most recent nationally representative sample of US adults, 100% fruit juice consumption was associated with better nutrient intake and better diet quality, and replacing 100% fruit juice with whole fruits equivalents resulted in only a limited impact on nutrient intake, except for a small increase in dietary fiber. 

Approximately 16% of the population consumed 100% fruit juice on the day of recall and the mean per capita usual intake was 0.26 cups equivalent per day. Although there are no specific recommendations for adults for 100% fruit juice consumption, DGA recognized one cup of 100% fruit juice as one cup serving of fruit and indicated that up to half the daily fruit intake may come from 100% juice in a healthy eating pattern [1]. The rationale for limiting 100% fruit juice intake to only half daily fruit intake was that the juice is lower in fiber than whole fruit [1]. In our dietary modeling study, isocaloric replacement of 100% fruit juice with whole fruit equivalents resulted in only a modest (6.4%) increase in usual intake of dietary fiber. An earlier modeling study conducted by USDA for the 2005 Dietary Guidelines Advisory Committee also reported improved fiber intake by replacing juices with fruit for children [35]. The Committee concluded that 100% fruit juice provided higher amounts of several important vitamins and minerals than whole fruits. However, we did not find any significant changes in the usual intakes as well as percentage of the population below the EAR/ above the AI of any other nutrients due to replacement of 100% fruit juice with whole fruit equivalents.

Consumers of 100% fruit juice had a better diet quality, as assessed by HEI-2015, in the present analysis. HEI is a validated marker of diet quality commonly used to evaluate diets and dietary interventions [36,37,38], to validate other nutrition research tools [39] and to understand relationships between nutrients/foods/dietary patterns and health-related outcomes [40,41,42]. A higher score of HEI-2015 is an indication of better compliance/adherence to key dietary recommendations of the DGA using 13 subcomponents (nine for adequacy and four for moderation) [1]. In the present analysis of NHANES 2013–2016 data, we found that the HEI-2015 total scores as well as subcomponent scores for total fruit, whole fruit, whole grain, sodium, saturated fat and added sugar of 100% fruit juice consumers were significantly higher than that those of non-consumers. A higher HEI-2015 score for total fruit, whole fruit and whole grain are indicative of their higher intakes while higher score for sodium, saturated fat and added sugars are indicative of their lower intakes [30]. These results are in agreement with earlier cross-sectional studies analyzing older versions of NHANES 2003–2006 [19,20] as well as other data sets [21,22]. In our present analysis, we additionally found a significant trend towards higher HEI 2015 score (total score and specific subcomponents scores) with increasing consumption of 100% fruit juice from <4 oz to >12 oz suggesting that diet quality increased with increasing 100% fruit juice intake. The fact that 100% fruit juice was also associated with increased sub-component scores for whole grain, sodium, and saturated fat suggests fruit juice consumers consume healthier foods/diets.

100% fruit juice consumers had significantly higher intake of calcium, magnesium, potassium, thiamin, folate, vitamins B6, vitamin C, vitamin D and beta-cryptoxanthin and intake of these nutrients (except thiamin) increased with increasing level of 100% fruit juice intake. Many of these nutrients are currently under-consumed and have been identified as “shortfall nutrients” by the DGA [1]. Additionally, the DGA has classified calcium, potassium, and vitamin D as “nutrients of public health concern” due to the fact that their current intakes are low enough to pose a public health concern [1]. Thus, foods containing these nutrients need to be promoted for children and adults. Similar improved intakes of many vitamin and minerals among 100% fruit juice consumers were also reported in earlier cross-sectional studies [19,20,21,22]. The consumers of 100% fruit juice had a 154 mg less sodium than non-consumers. High sodium intake has been linked to blood pressure and therefore limiting dietary sodium is an important public health improvement target [1]. The consumers of 100% fruit juice also had a higher energy intake and higher intake total sugar than non-consumers in the present analysis. However, the intake of added sugars was significantly lower in 100% fruit juice consumers, indicating that consumers are probably not consuming as much sugar sweetened beverages. Although 100% fruit juice contains naturally occurring sugars, it has no added sugar. DGA also recommended limiting added sugar to 10% total daily energy intake [1].

Additionally, adult consumers of 100% fruit juice also had lower BMI/body weight and certain metabolic markers, and a reduced risk for obesity and metabolic syndrome. Consumers of 100% orange juice also had lower BMI and cardiometabolic markers in earlier analysis with NHANES 1999–2004 and 2003–2006 [20,43,44], and another database [45]. However, some cross-sectional studies reported no association between 100% fruit juice and BMI among French adults [22], or a positive association among postmenopausal women [46]. It is interesting to note that although compared to non-consumers, 100% fruit juice consumers had 8% more energy intake on the day of the recall, they had about 4% lower BMI/body weight and were at 22% less risk for being overweight/obese in the present analysis. However, as noted above, juice consumers had better diet quality (10% higher HEI-2015 score) than non-consumers. Diet quality may play a significant role in body weight metabolism. However, more research especially using randomized controlled trials are needed to confirm this. 

A major limitation of this study is the use of cross-sectional study design, which cannot be used to determine cause and effect. The dietary intake data were self-reported recalls relying on memory, and are potentially subject to reporting bias. While dietary recalls in NHANES were collected using one of the best available and validated methodology, the AMPM method, there are still limitations with it [47]. Finally, a single 24-h recall only provides consumption patterns of the day of recall and may not be sufficient to separate regular consumers from non-consumers [48]. It is also important to recognize that the results from this study do not specifically reflect the effect of fruit juice consumption only, but rather reflect the consumption of fruit juice within the context of the total diet. While we used a number of covariates to adjust our results, we cannot rule out that residual confounding may explain some of the reported associations.

## 5. Conclusions

Results from this study show that the consumption of 100% fruit juice was associated with better nutrient intake and diet quality and the association was also related to the consumption level. Isocaloric replacement of 100% fruit juice with whole fruits equivalents had no effect on nutrient intake, except for a small increase in dietary fiber.

## Figures and Tables

**Table 1:** Food codes of 100% fruit juices and of whole fruit equivalents used for 100% fruit juice replacement.

100% Fruit Juice		Whole Fruit Equivalent	
Food Code	Description	Food Code	Description
61201010	Grapefruit juice, 100%, freshly squeezed	61101010	Grapefruit, raw
61201020	Grapefruit juice, 100%, NS as to form		
61201220	Grapefruit juice, 100%, canned, bottled or in a carton		
61201225	Grapefruit juice, 100%, with calcium added		
61210000	Orange juice, 100%, NFS	61119010	Orange, raw
61210010	Orange juice, 100%, freshly squeezed		
61210220	Orange juice, 100%, canned, bottled or in a carton		
61210250	Orange juice, 100%, with calcium added, canned, bottled or in a carton		
61210620	Orange juice, 100%, frozen, reconstituted		
61210820	Orange juice, 100%, with calcium added, frozen, reconstituted		
61213220	Tangerine juice, 100%	61125010	Tangerine, raw
61213800	Fruit juice blend, citrus, 100% juice	63311000	Fruit salad, fresh or raw, excluding citrus fruits, no dressing
64100100	Fruit juice, NFS		
64100110	Fruit juice blend, 100% juice		
64100200	Cranberry juice blend, 100% juice	63207010	Cranberries, raw
64100220	Cranberry juice blend, 100% juice, with calcium added		
64101010	Apple cider	63101000	Apple, raw
64104010	Apple juice, 100%		
64104030	Apple juice, 100%, with calcium added		
64104600	Blackberry juice, 100%	63201010	Blackberries, raw
64105400	Cranberry juice, 100%, not a blend	63207010	Cranberries, raw
64116020	Grape juice, 100%	63123000	Grapes, raw, NS as to type
64116060	Grape juice, 100%, with calcium added		
64120010	Papaya juice, 100%	63133010	Papaya, raw
64121000	Passion fruit juice, 100%	63134010	Passion fruit, raw
64124020	Pineapple juice, 100%	63141010	Pineapple, raw
64126000	Pomegranate juice, 100%	63145010	Pomegranate, raw
64132010	Prune juice, 100%	63143010	Plum, raw
64132500	Strawberry juice, 100%	63223020	Strawberries, raw
64133100	Watermelon juice, 100%	63149010	Watermelon, raw

**Table 2:** Demographics associated with 100% fruit juice consumption in adults (19+ years of age)—NHANES 2013–2016 *.

Variables	Non-Consumers	Consumers	*P* Value	100% Fruit Juice Consumption Levels (Oz/Day)			
				>0 to 4	>4 to 8	>8 to 12	>12
Population (%)	84.4 ± 0.5	15.6 ± 0.5		1.16 ± 0.15	4.65 ± 0.31	4.16 ± 0.20	5.67 ± 0.31
Age (years)	47.3 ± 0.4	49.8 ± 0.7	0.0009	53.3 ± 2.2	55.6 ± 1.1	49.4 ± 1.0	44.4 ± 1.2
Gender (% Male)	48.8 ± 0.7	52.6 ± 1.5	0.0469	28.5 ± 6.0	46.8 ± 2.8	54.9 ± 3.1	60.7 ± 2.3
Ethnicity							
Hispanic (%)	14.6 ± 1.7	17.3 ± 1.9	0.0077	13.9 ± 3.6	11.2 ± 2.1	17.4 ± 2.6	23.0 ± 3.0
Non-Hispanic White (%)	65.6 ± 2.4	61.1 ± 2.9	0.0043	63.1 ± 6.4	72.9 ± 3.6	58.8 ± 3.9	52.7 ± 3.2
Non-Hispanic Black (%)	10.6 ± 1.3	14.8 ± 1.7	<0.0001	15.1 ± 4.0	10.8 ± 2.1	16.5 ± 2.4	16.8 ± 1.8
Asian (%)	5.72 ± 0.80	4.76 ± 0.79	0.0505	5.07 ± 1.78	4.30 ± 0.98	4.86 ± 1.19	4.99 ± 0.88
Other (%)	3.50 ± 0.39	2.00 ± 0.37	0.0079	2.79 ± 1.49	0.81 ± 0.39	2.43 ± 0.84	2.51 ± 0.58
Physical Activity							
Sedentary (%)	22.1 ± 0.8	21.0 ± 1.6	0.5052	27.5 ± 6.3	25.2 ± 2.7	21.7 ± 2.9	15.7 ± 1.8
Moderate (%)	35.2 ± 0.7	38.2 ± 1.9	0.1364	40.7 ± 6.2	39.9 ± 2.9	38.3 ± 2.8	36.2 ± 3.2
Vigorous (%)	42.7 ± 1.0	40.8 ± 1.9	0.3296	31.8 ± 5.5	34.9 ± 3.0	39.9 ± 3.0	48.1 ± 3.0
Poverty Income Ratio							
<1.35 (%)	23.7 ± 1.5	24.6 ± 2.4	0.6397	23.6 ± 5.9	19.3 ± 2.8	24.9 ± 2.8	28.9 ± 3.4
1.35–1.85 (%)	10.2 ± 0.7	11.3 ± 1.2	0.4042	4.1 ± 1.7	13.7 ± 2.3	10.4 ± 1.7	11.6 ± 1.7
>1.85	66.1 ± 1.9	64.1 ± 2.8	0.3324	72.3 ± 6.4	67.0 ± 3.5	64.7 ± 3.1	59.5 ± 3.6
Smoking Current (% Yes)	20.2 ± 0.9	11.8 ± 1.3	<0.0001	8.2 ± 2.9	10.5 ± 2.2	10.5 ± 1.6	14.5 ± 1.8
Obese (%)	39.5 ± 1.0	34.2 ± 1.8	0.0111	27.0 ± 4.8	32.1 ± 3.3	32.3 ± 2.4	38.9 ± 3.0
Overweight (%)	32.2 ± 0.6	34.4 ± 1.3	0.1485	44.4 ± 7.4	34.9 ± 2.9	39.7 ± 2.3	28.0 ± 2.4
Body Mass Index (kg/m^2^)	29.4 ± 0.2	28.4 ± 0.3	0.0027	28.1 ± 0.6	28.2 ± 0.5	28.7 ± 0.4	28.5 ± 0.5

* Data is presented as Mean ± Standard Error (SE).

**Table 3:** Energy and nutrients intake associated with 100% fruit juice consumption in adults (19+ years of age, *n* = 9152)—NHANES 2013–2016 *.

	Non-Consumers	Consumers	*P* Value	100% Fruit Juice Consumption Levels (Oz/Day)				
				>0 to 4	>4 to 8	>8 to 12	>12	*P*_group trend_
Energy (kcal)	2088 ± 11	2262 ± 31	<0.0001	2021 ± 74	2198 ± 63	2267 ± 62	2366 ± 45	<0.0001
Carbohydrate (g)	243 ± 1	264 ± 2	<0.0001	241 ± 5	263 ± 4	260 ± 3	272 ± 4	<0.0001
Total sugars (g)	105 ± 1	124 ± 2	<0.0001	95.2 ± 5.2	120 ± 4	123 ± 3	134 ± 3	<0.0001
Added sugars (tsp eq)	17.2 ± 0.03	14.7 ± 0.4	<0.0001	13.8 ± 1.1	17.0 ± 1.0	15.4 ± 0.6	12.5 ± 0.7	<0.0001
Dietary fiber (g)	17.0 ± 0.2	17.3 ± 0.3	0.4661	16.7 ± 0.8	17.9 ± 0.6	16.3 ± 0.4	17.6 ± 0.5	0.4908
Total fat (g)	84.2 ± 0.4	76.7 ± 0.9	<0.0001	85.8 ± 2.0	77.3 ± 1.3	77.2 ± 1.2	73.8 ± 1.6	<0.0001
Cholesterol (mg)	296 ± 3	289 ± 8	0.4742	317 ± 16	284 ± 1.3	274 ± 13	300 ± 17	0.5822
Protein (g)	83.0 ± 0.6	79.8 ± 0.7	0.0024	80.9 ± 2.1	79.4 ± 1.8	82.4 ± 2.0	78.0 ± 1.9	0.0050
Calcium (mg)	940 ± 8	1015 ± 11	<0.0001	840 ± 37	948 ± 31	1083 ± 31	1059 ± 24	<0.0001
Iron (mg)	14.1 ± 0.1	14.5 ± 0.3	0.3345	14.1 ± 0.7	15.1 ± 0.5	14.6 ± 0.4	13.9 ± 0.4	0.5709
Magnesium (mg)	302 ±3	312 ± 4	0.0285	297 ± 10	307 ± 7	313 ± 9	320 ± 8	0.0163
Phosphorus (mg)	1384 ± 9	1370 ± 10	0.2844	1310 ± 22	1372 ± 24	1402 ± 28	1358 ± 28	0.4451
Potassium (mg)	2578 ± 19	2918 ± 28	<0.0001	2606 ± 79	2749 ± 55	2886 ± 65	3158 ± 50	<0.0001
Sodium (mg)	3540 ± 17	3386 ± 41	0.0014	3842 ± 255	3412 ± 62	3398 ± 96	3251 ± 65	<0.0001
Vitamin A, RAE (µg)	626 ± 9	640 ± 19	0.5025	708 ± 82	660 ± 39	691 ± 21	570 ± 30	0.9135
Thiamin (Vitamin B1) (mg)	1.58 ± 0.01	1.66 ± 0.02	0.0124	1.54 ± 0.05	1.64 ± 0.04	1.70 ± 0.04	1.66 ± 0.05	0.0135
Riboflavin (Vitamin B2) (mg)	2.17 ± 0.01	2.13 ± 0.03	0.1409	2.15 ± 0.12	2.17 ± 0.06	2.24 ± 0.07	2.00 ± 0.05	0.0418
Niacin (mg)	26.2 ± 0.2	25.4 ± 0.4	0.0831	25.3 ± 1.2	25.7 ± 0.6	27.1 ± 0.8	23.8 ± 0.8	0.0461
Folate, DFE (µg)	507 ± 6	558 ± 14	0.0024	493 ± 27	574 ± 27	558 ± 18	558 ± 21	0.0021
Vitamin B6 (mg)	2.13 ± 0.02	2.27 ± 0.05	0.0115	2.15 ± 0.13	2.22 ± 0.08	2.40 ± 0.09	2.23 ± 0.08	0.0147
Vitamin C (mg)	64.3 ± 1.4	156 ± 4	<0.0001	77.6 ± 3.1	112 ± 5	145 ± 4	218 ± 6	<0.0001
Vitamin D (D2 + D3) (µg)	4.56 ± 0.09	5.37 ± 0.35	0.0308	4.55 ± 0.39	5.05 ± 0.53	5.51 ± 0.45	5.73 ± 0.78	0.0406
Vitamin E as α -tocopherol (mg)	9.28 ± 13	9.08 ± 0.26	0.4773	9.26 ± 0.39	9.22 ± 0.43	9.35 ± 044	8.72 ± 0.60	0.4171
Total choline (mg)	336 ± 2	338 ± 6	0.7196	336 ± 16	333 ± 7	330 ± 10	351 ± 15	0.5612
Beta-carotene (mcg)	2207 ± 73	2359 ± 166	0.3958	3041 ± 575	2634 ± 349	2456 ± 216	1896 ± 256	0.9688
Beta-cryptoxanthin (µg)	77.9 ± 3.2	133 ± 6	<0.0001	85.5 ± 12.9	98.3 ± 7.5	132 ± 11	173 ± 11	<0.0001
Lycopene (µg)	5118 ± 167	4743 ± 319	0.3470	4902 ± 898	4981 ± 446	4632 ± 484	4587 ± 618	0.3229
Lutein + zeaxanthin (µg)	1603 ± 69	1696 ± 99	0.4927	1926 ± 571	1510 ± 126	1698 ± 159	1802 ± 234	0.4134

* Data adjusted for age, gender, ethnicity, physical activity level, poverty income ratio level, smoking current status, alcohol and kcal (except for energy); and presented as Least Square Mean (LSM) ± Standard Error (SE).

**Table 4:** Healthy Eating Index (HEI) 2015) and sub-component scores associated with 100% fruit juice consumption in adults (19+ years of age, *n* = 9152)—NHANES 2013–2016 *.

HEI 2015 Components	Non-Consumers	Consumers	*P* Value	100% Fruit Juice Consumption Levels (Oz/Day)				
				>0 to 4	>4 to 8	>8 to 12	>12	*P*_group trend_
Total score	50.4 ± 0.3	55.4 ± 0.4	<0.0001	51.9 ± 0.1.9	54.9 ± 0.8	54.3 ± 0.9	57.4 ± 0.6	<0.0001
Component 1 (total vegetables)	3.07 ± 0.03	3.05 ± 0.06	0.7784	3.22 ± 0.22	3.24 ± 0.12	2.93 ± 0.11	2.93 ± 0.09	0.3500
Component 2 (greens and beans)	1.62 ± 0.05	1.67 ± 0.11	0.6911	1.23 ± 0.28	1.68 ± 0.18	1.73 ± 0.16	1.72 ± 0.14	0.5074
Component 3 (total fruit)	1.63 ± 0.05	4.03 ± 0.04	<0.0001	2.63 ± 0.23	3.53 ± 0.11	4.02 ± 0.07	4.76 ± 0.04	<0.0001
Component 4 (whole fruit)	2.03 ± 0.05	2.35 ± 0.09	0.0007	2.04 ± 0.34	2.59 ± 0.18	2.19 ± 0.14	2.34 ± 0.14	0.0015
Component 5 (whole grains)	2.61 ± 0.04	2.95 ± 0.13	0.0125	3.91 ± 0.39	3.00 ± 0.23	2.94 ± 0.24	2.69 ± 0.18	0.0513
Component 6 (dairy)	5.05 ± 0.06	4.92 ± 0.09	0.2091	4.32 ± 0.46	5.04 ± 0.22	5.24 ± 0.24	4.71 ± 0.17	0.2301
Component 7 (total protein foods)	4.25 ± 0.02	4.17 ± 0.04	0.1083	4.40 ± 0.14	4.17 ± 0.07	4.09 ± 0.09	4.18 ± 0.07	0.0665
Component 8 (seafood and plant protein)	2.40 ± 0.05	2.34 ± 0.09	0.5048	2.06 ± 0.26	2.28 ± 0.13	2.13 ± 0.15	2.61 ± 0.15	0.9908
Component 9 (fatty acid ratio)	5.04 ± 0.07	4.93 ± 0.12	0.4197	5.06 ± 0.51	5.17 ± 0.27	4.67 ± 0.27	4.89 ± 0.24	0.2886
Component 10 (sodium)	4.08 ± 0.05	4.79 ± 0.12	<0.0001	3.94 ± 0.61	4.53 ± 0.18	4.80 ± 0.28	5.20 ± 0.18	<0.0001
Component 11 (refined grain)	6.26 ± 0.06	6.49 ± 0.14	0.1517	5.87 ± 0.35	6.37 ± 0.26	6.22 ± 0.25	6.94 ± 0.24	0.0520
Component 12 (saturated fat)	5.62 ± 0.06	6.38 ± 0.13	<0.0001	5.37 ± 0.36	6.44 ± 0.20	6.23 ± 0.24	6.67 ± 0.21	<0.0001
Component 13 (added sugar)	6.72 ± 0.06	7.35 ± 0.11	<0.0001	7.83 ± 0.49	6.90 ± 0.19	7.15 ± 0.17	7.77 ± 0.20	<0.0001

* Data adjusted for age, gender, ethnicity, physical activity level, poverty income ratio level, smoking current status, and alcohol; and presented as Least Square Mean (LSM) ± Standard Error (SE).

**Table 5:** Effect of isocaloric replacement of 100% fruit juice with whole fruit equivalents on usual intakes of nutrients and population adequacy for adult (19+ years of age, *n* = 10,112)—NHANES 2013–2016 *.

	Baseline, No Replacement	After Replacement	*P* Value (z stat) ^1^	Baseline No Replacement	After Replacement	*P* Value (z stat)
**Nutrients with EAR**	**Usual Intakes**			**% Adults Below EAR**		
Calcium (mg)	958 ± 9	950 ± 9	0.4985	44.5 ± 1.0	45.2 ± 1.0	0.6273
Carbohydrate (g)	249 ± 1	249 ± 1	0.8444	1.17 ± 0.17	1.15 ± 0.17	0.9556
Folate, DFE (µg)	523 ± 5	526 ± 5	0.6513	14.6 ± 0.9	14.1 ± 0.9	0.7338
Iron (mg)	14.3 ± 0.1	14.4 ± 0.1	0.9657	5.99 ± 0.32	6.05 ± 0.35	0.9093
Magnesium (mg)	305 ± 3	305 ± 3	0.8929	53.5 ± 1.1	53.6 ± 1.1	0.919
Niacin (mg)	26.3 ± 0.2	26.3 ± 0.2	0.9296	1.63 ± 0.22	1.63 ± 0.21	0.9969
Phosphorus (mg)	1393 ± 10	1390 ± 10	0.8442	0.74 ± 0.14	0.76 ± 0.15	0.9214
Protein (g)	83.1 ± 0.6	83.2 ± 0.6	0.8694	1.97 ± 0.27	1.97 ± 0.26	0.9987
Riboflavin (Vitamin B2) (mg)	2.17 ± 0.02	2.18 ± 0.02	0.8926	3.23 ± 0.3	3.17 ± 0.29	0.8786
Thiamin (Vitamin B1) (mg)	1.61 ± 0.01	1.62 ± 0.01	0.3465	7.78 ± 0.7	7.45 ± 0.7	0.7411
Vitamin A, RAE (µg)	633 ± 8	638 ± 8	0.6554	45.7 ± 1.1	44.9 ± 1.2	0.6217
Vitamin B6 (mg)	2.16 ± 0.02	2.16 ± 0.02	0.9752	11.7 ± 0.7	11.6 ± 0.7	0.9284
Vitamin C (mg)	79.4 ± 1.4	83.3 ± 1.5	0.0599	48.0 ± 1.4	46.3 ± 1.4	0.3767
Vitamin D (D2 + D3) (µg)	4.68 ± 0.08	4.59 ± 0.08	0.4371	94.9 ± 0.6	95.3 ± 0.5	0.5733
Vitamin E as alpha-tocopherol (mg)	9.25 ± 0.13	9.33 ± 0.13	0.6416	79.0 ± 1.2	78.4 ± 1.2	0.729
**Nutrients with AI**	**Usual Intakes**			**% Adults Above AI**		
Dietary fiber (g)	17.1 ± 0.2	18.2 ± 0.2	0.0008	7.93 ± 0.67	10.5 ± 0.8	0.0102
Potassium (mg)	2644 ± 21	2650 ± 21	0.8212	1.73 ± 0.21	1.77 ± 0.21	0.8887
Sodium (mg)	3539 ± 24	3534 ± 24	0.9024	99.5 ± 0.1	99.5 ± 0.1	0.987
Total choline (mg)	339 ± 2	340 ± 3	0.6914	8.42 ± 0.72	8.58 ± 0.72	0.8777

* Data presented as Mean ± Standard Error (SE). EAR—Estimated Average Requirement; AI—Adequate Intake; ^1^ Z-statistic was used to assess difference in baseline and replacement of whole fruit for fruit juice by comparing Z-statistic to a normal distribution table.

## References

[1] U.S. Department of Health, Human Services, U.S. Department of Agriculture 2015–2020 Dietary Guidelines for Americans. http://health.gov/dietaryguidelines/2015/guidelines/.

[2] Joint WHO/FAO Expert Consultation (2003). Diet, Nutrition and the Prevention of Chronic Diseases.

[3] Aune D., Giovannucci E., Boffetta P., Fadnes L.T., Keum N., Norat T., Greenwood D.C., Riboli E., Vatten L.J., Tonstad S. (2017). Fruit and vegetable intake and the risk of cardiovascular disease, total cancer and all-cause mortality-a systematic review and dose-response meta-analysis of prospective studies. Int. J. Epidemiol..

[4] Wang X., Ouyang Y., Liu J., Zhu M., Zhao G., Bao W., Hu F.B. (2014). Fruit and vegetable consumption and mortality from all causes, cardiovascular disease, and cancer: Systematic review and dose-response meta-analysis of prospective cohort studies. BMJ.

[5] Yip C.S.C., Chan W., Fielding R. (2019). The Associations of Fruit and Vegetable Intakes with Burden of Diseases: A Systematic Review of Meta-Analyses. J. Acad. Nutr. Diet..

[6] USDA (2018). Choose My Plate: All about the Fruit Group. https://www.choosemyplate.gov/fruit.

[7] Liu R.H. (2013). Health-promoting components of fruits and vegetables in the diet. Adv. Nutr..

[8] Slavin J.L., Lloyd B. (2012). Health Benefits of fruits and vegetables. Adv. Nutr..

[9] World Health Organization (2009). Global Health Risks: Mortality and Burden of Disease Attributable to Selected Major Risks.

[10] Office of Disease Prevention and Health Promotion (2018). Using Law and Policy to Increase Fruit and Vegetable Intake in the United States. https://www.healthypeople.gov/sites/default/files/NWS_ExecutiveSummary_2018-10.03.pdf.

[11] Lee-Kwan S.H., Moore L.V., Blanck H.M., Harris D.M., Galuska D. (2017). Disparities in State-Specific Adult Fruit and Vegetable Consumption—United States, 2015. MMWR.

[12] Wojcicki J.M., Heyman M.B. (2012). Reducing childhood obesity by eliminating 100% fruit juice. Am. J. Public Health.

[13] Shefferly A., Scharf R.J., DeBoer M.D. (2016). Longitudinal evaluation of 100% fruit juice consumption on BMI status in 2–5-year-old children. Pediatr. Obes..

[14] Guasch-Ferre M., Hu F.B. (2019). Are Fruit Juices Just as Unhealthy as Sugar-Sweetened Beverages?. JAMA Netw. Open.

[15] Crowe-White K., O’Neil C.E., Parrott J.S., Benson-Davies S., Droke E., Gutschall M., Stote K.S., Wolfram T., Ziegler P. (2016). Impact of 100% Fruit Juice Consumption on Diet and Weight Status of Children: An Evidence-based Review. Crit. Rev. Food Sci. Nutr..

[16] Auerbach B.J., Wolf F.M., Hikida A., Vallila-Buchman P., Littman A., Thompson D., Louden D., Taber D.R., Krieger J. (2017). Fruit Juice and Change in BMI: A Meta-analysis. Pediatrics.

[17] Liu K., Xing A., Chen K., Wang B., Zhou R., Chen S., Xu H., Mi M. (2013). Effect of fruit juice on cholesterol and blood pressure in adults: A meta-analysis of 19 randomized controlled trials. PLoS ONE.

[18] Murphy M.M., Barrett E.C., Bresnahan K.A., Barraj L.M. (2017). 100 % Fruit juice and measures of glucose control and insulin sensitivity: A systematic review and meta-analysis of randomized controlled trials. J. Nutr. Sci..

[19] O’Neil C.E., Nicklas T.A., Zanovec M., Fulgoni V.L. (2011). Diet quality is positively associated with 100% fruit juice consumption in children and adults in the United States: NHANES 2003-2006. Nutr. J..

[20] O’Neil C.E., Nicklas T.A., Rampersaud G.C., Fulgoni V.L. (2011). 100% Orange juice consumption is associated with better diet quality, improved nutrient adequacy, decreased risk for obesity, and improved biomarkers of health in adults: National Health and Nutrition Examination Survey, 2003-2006. Nutr. J..

[21] Francou A., Hebel P., Braesco V., Drewnowski A. (2015). Consumption Patterns of Fruit and Vegetable Juices and Dietary Nutrient Density among French Children and Adults. Nutrients.

[22] Bellisle F., Hebel P., Fourniret A., Sauvage E. (2018). Consumption of 100% Pure Fruit Juice and Dietary Quality in French Adults: Analysis of a Nationally Representative Survey in the Context of the WHO Recommended Limitation of Free Sugars. Nutrients.

[23] Nicklas T.A., O’Neil C.E., Fulgoni V.L. (2015). Consumption of 100% Fruit Juice is Associated with Better Nutrient Intake and Diet Quality but not with Weight Status in Children: NHANES 2007-2010. Int. J. Child Health Nutr..

[24] O’Neil C.E., Nicklas T.A., Zanovec M., Kleinman R.E., Fulgoni V.L. (2012). Fruit juice consumption is associated with improved nutrient adequacy in children and adolescents: The National Health and Nutrition Examination Survey (NHANES) 2003-2006. Public Health Nutr..

[25] O’Neil C.E., Nicklas T.A., Kleinman R.E. (2010). Relationship between 100% juice consumption and nutrient intake and weight of adolescents. Am. J. Health Promot..

[26] Centers for Disease Control and Prevention (CDC), National Center for Health Statistics (2019). National Health and Nutrition Examination Survey.

[27] Raper N., Perloff B., Ingwersen L., Steinfeldt L., Anand J. (2004). An overview of USDA’s dietary intake data system. J. Food Comp. Anal..

[28] US Department of Agriculture National Agriculture Library: National Nutrient Database for Standard Reference. http://ndb.nal.usda.gov/.

[29] U.S. Department of Agriculture, Agricultural Research Service (2018). USDA Food and Nutrient Database for Dietary Studies. Food Surveys Research Group Home Page. http://www.ars.usda.gov/nea/bhnrc/fsrg.

[30] Krebs-Smith S.M., Pannucci T.E., Subar A.F., Kirkpatrick S.I., Lerman J.L., Tooze J.A., Wilson M.M., Reedy J. (2018). Update of the Healthy Eating Index: HEI-2015. J. Acad. Nutr. Diet..

[31] Matthews D.R., Hosker J.P., Rudenski A.S., Naylor B.A., Treacher D.F., Turner R.C. (1985). Homeostasis model assessment: Insulin resistance and beta-cell function from fasting plasma glucose and insulin concentrations in man. Diabetologia.

[32] National Institutes of Health (2002). National Cholesterol Education Program: National heart, lung, and blood institute. Detection, Evaluation, and Treatment of High Blood Cholesterol in Adults (Adult Treatment Panel III).

[33] National Institutes of Health: National Heart, Lung, and Blood Institute Clinical Guidelines on the Identification, Evaluation, and Treatment of Overweight and Obesity in Adults. http://www.nhlbi.nih.gov/guidelines/obesity/ob_gdlns.pdf.

[34] Tooze J.A., Kipnis V., Buckman D.W., Carroll R.J., Freedman L.S., Guenther P.M., Krebs-Smith S.M., Subar A.F., Dodd K.W. (2010). A mixed-effects model approach for estimating the distribution of usual intake of nutrients: The NCI method. Stat. Med..

[35] US Department of Agriculture (2004) 2005 Dietary Guidelines Advisory Committee Report. Fruit and Fruit Juice Analysis. http://www.health.gov/dietaryguidelines/dga2005/report/HTML/G2_Analyses.htm#fruitjuice.

[36] Hiza H.A., Casavale K.O., Guenther P.M., Davis C.A. (2013). Diet quality of Americans differs by age, sex, race/ethnicity, income, and education level. J. Acad. Nutr. Diet..

[37] Reedy J., Krebs-Smith S.M., Bosire C. (2010). Evaluating the food environment: Application of the Healthy Eating Index-2005. Am. J. Prev. Med..

[38] Juan W.Y., Guenther P.M., Kott P.S. (2008). Nutrition Insight 41. Diet Quality of Older Americans in 1994–96 and 2001–02 as Measured by the Healthy Eating Index-2005.

[39] Fulgoni V.L., Keast D.R., Drewnowski A. (2009). Development and validation of the Nutrient-rich Foods Index: A tool to measure nutritional quality of foods. J. Nutr..

[40] Nicklas T.A., O’Neil C.E., Fulgoni V.L. (2012). Diet quality is inversely related to cardiovascular risk factors in adults. J. Nutr..

[41] Chiuve S., Fung T., Rimm E., Hu F., McCullough M., Wang M., Stampfer M.J., Willett W.C. (2012). Alternative dietary indices both strongly predict risk of chronic disease. J. Nutr..

[42] Reedy J., Mitrou P.N., Krebs-Smith S.M., Wirfält E., Flood A., Kipnis V., Leitzmann M., Mouw T., Hollenbeck A., Schatzkin A. (2008). Index-based dietary patterns and risk of colorectal cancer: The NIH-AARP Diet and Health Study. Am. J. Epidemiol..

[43] Pereira M.A., Fulgoni V.L. (2010). Consumption of 100% fruit juice and risk of obesity and metabolic syndrome: Findings from the national health and nutrition examination survey 1999–2004. J. Am. Coll. Nutr..

[44] Wang Y., Lloyd B., Yang M., Davis C.G., Lee S.G., Lee W., Chung S.J., Chun O.K. (2012). Impact of orange juice consumption on macronutrient and energy intakes and body composition in the US population. Public Health Nutr..

[45] Akhtar-Danesh N., Dehghan M. (2010). Association between fruit juice consumption and self-reported body mass index among adult Canadians. J. Hum. Nutr Diet..

[46] Auerbach B.J., Littman A.J., Krieger J., Young B.A., Larson J., Tinker L., Neuhouser M.L. (2018). Association of 100% fruit juice consumption and 3-year weight change among postmenopausal women in the in the Women’s Health Initiative. Prev. Med..

[47] Bodner-Montville J., Ahuja J., Ingwersen L.A., Haggertyt E.S., Enns C.W., Perloff B.P. (2006). USDA Food and Nutrient Database for Dietary Studies: Released on the Web. J. Food Compos. Anal..

[48] Ahluwalia N., Dwyer J., Terry A., Moshfegh A., Johnson C. (2016). Update on NHANES dietary data: Focus on collection, release, analytical considerations, and uses to inform public policy. Adv. Nutr..

